# The Epidemiology of *Staphylococcus aureus* and Panton-Valentine Leucocidin (*pvl*) in Central Australia, 2006-2010

**DOI:** 10.1186/s12879-016-1698-5

**Published:** 2016-08-08

**Authors:** S. Hewagama, T. Spelman, M. Woolley, J. McLeod, D. Gordon, L. Einsiedel

**Affiliations:** 1 Alice Springs Hospital, Alice Springs, NT Australia; 2 Flinders University/Northern Territory Rural Clinical School, Alice Springs, NT Australia; 3 SA Pathology, Adelaide, Australia; 4 NT Pathology, Alice Springs, NT Australia; 5 Baker IDI Heart and Diabetes Institute, Alice Springs, NT Australia

**Keywords:** Staphylococcus aureus, Panton-Valentine Leucocidin, ST93

## Abstract

**Background:**

The Central Australian Indigenous population has a high incidence of *Staphylococcus aureus* bacteremia (SAB) but little is known about the local molecular epidemiology.

**Methods:**

Prospective observational study of bacteremic and nasal colonizing *S.*aureus isolates between June 2006 to June 2010. All isolates underwent single nucleotide polymorphism (SNP) genotyping and testing for the presence of the Panton-Valentine Leucocidin (*pvl*) gene.

**Results:**

Invasive isolates (*n* = 97) were predominantly ST93 (26.6 %) and *pvl* positive (54.3 %), which was associated with skin and soft tissue infections (OR 4.35, 95 % CI 1.16, 16.31). Non-multiresistant MRSA accounted for 31.9 % of bacteremic samples and showed a trend to being healthcare associated (OR 2.16, 95 % CI 0.86, 5.40). Non-invasive isolates (*n* = 54) were rarely ST93 (1.9 %) or pvl positive (7.4 %).

**Conclusions:**

In Central Australia, ST93 was the dominant *S.aureus* clone, and was frequently *pvl* positive and associated with an aggressive clinical phenotype. Whether non-nasal carriage is more important with invasive clones or whether colonization occurs only transiently remains to be elucidated.

## Background

*Staphylococcus aureus* remains one of the most clinically important bacteria. Approximately 60 % of humans are transiently colonized and up to 30 % remain colonized lifelong [1, 2]. Colonization conveys a significant risk of invasive disease [1] including *S.aureus* bacteremia (SAB), which continues to carry a significant risk of death [3].

Methicillin resistance in *S.aureus* is conveyed by the *mecA* gene, found on mobile genetic elements known as the staphylococcal chromosomal cassette (SCC*mec*) [2]. Initially, methicillin resistance was restricted to healthcare associated organisms (HA-MRSA) amongst SCC*mec* types I-III, but community acquired methicillin resistant S.aureus (CA-MRSA) have recently emerged (SCC*mec* IV or V). Such isolates often remain susceptible to non-beta-lactam antibiotics [2], hence their description as non-multiresistant (nm) MRSA. Although CA-MRSA were first reported in remote Australian Aboriginal communities [4], strains have now emerged independently worldwide. More recently, strains that express the Panton-Valentine leukocidin (*pvl*) gene show a propensity for skin and soft tissue infections (SSTI), especially furunculosis, and less commonly necrotizing pneumonia. Whether *pvl* toxin directly results in such presentations or is simply a virulence marker remains unclear. Furthermore, *pvl* is not restricted to MRSA [3].

*S.aureus* can be subtyped by multilocus sequence typing (MLST), by SCC*mec* gene and by the presence of *pvl*. Although different subtypes vary regionally, the genetically unique and highly virulent ST93 now predominates in Australia [5], a situation that is analogous to the dominance of USA300 in the USA [2].

Central Australia is an isolated region inhabited by a socially disadvantaged Indigenous population, mostly residing in isolated remote communities and to a lesser extent, in ‘town camps’ within the central town of Alice Springs. The non-Indigenous population predominantly lives in Alice Springs. The Indigenous population has among the highest incidence rates of SAB reported, 160.7 per 100,000 persons with more than 50 % attributable to SSTI and osteoarticular infections, approximately 20 % due to CA-MRSA [6], but the relative contribution of different MLST types and *pvl* are unknown. Given that methicillin resistance is common, efforts to isolate MRSA colonized patients within the hospital is standard practice. Whereas nasal colonization may play a role in HA-MRSA infections [1], its relevance in CA-MRSA is uncertain because strains often colonize sites other than the nasopharynx [3].

This study aimed to describe the molecular epidemiology of *S.aureus* in Central Australia and the rates of *pvl* amongst; (i) strains found on routine nasal screening and (ii) amongst bacteremic isolates.

## Methods

We prospectively obtained all SAB isolates at Alice Springs Hospital (ASH) between 1st June 2006 and 31st January 2010 and separately collected all routinely performed intensive care unit screening nasal swabs performed between 1st May 2009 and 30th June 2010 where *S.aureus* was cultured. As per ASH admission criteria, those aged less than 14 years old were classified as pediatric.

Identification was initially by latex agglutination and DNAse testing. Antibiotic susceptibility was determined by Vitek (bioMerieux). MRSA was identified by cefoxitin resistance and nmMRSA was defined as an organism that was methicillin resistant but retained sensitivity to 3 or more non-beta-lactam antibiotics including gentamicin, trimethoprim-sulfamethoxazole, doxycycline, erythromycin, clindamycin, rifampicin, fusidic acid and ciprofloxacin [4].

*S.aureus* isolates were grown on horse-blood agar plates overnight at 37 °C. Five colonies from cultures were suspended in 180 μl of 200 μg/ml Lysostaphin enzyme solution, incubated at 37 °C for 30 minutes, before DNA was extracted using a QIAGEN QIAamp DNA Mini kit. Methicillin resistance was confirmed by the presence of the *mecA* gene, as determined by PCR [7].

Staphylococcal sequence types were determined by identifying sets of single nucleotide polymorphisms (SNPs) that have been previously demonstrated to differentiate *Staphylococcus aureus* strains into approximately 27 genotypes. These sequence types are consistent with those identified by the more involved multilocus sequence typing (MLST) and eBURST analysis [8], an algorithm that displays the likely pattern of genomic evolution of bacterial organisms [9]. “The “Minimum SNPs” program [10] was used to calculate SNP sets, from data held within the *S.aureus* MLST database, optimized through maximization of the Simpson’s index of diversity. SNP genotyping was performed using the single tube kinetic PCR (STKP) method [11] that allows for the examination of multiple targets using a generic mix of primers, streamlined into a single tube reaction [11]. The conventional “two-tube” interrogation was applied to verify allele-specific real-time PCR cycle times for *S.aureus* sequence type (ST) 1 [11] and for ambiguous SNP results. Thereafter the STKP method was employed to interrogate each *S.aureus* isolate. MLST was performed on selected isolates for ST and SNP profiling verification [12].

The ‘Queensland clone’, ST93, is a singleton by eburst analysis. The ST93 SNP profile is shared by clonal complexes ST59 and ST121 and other singletons. Minimum SNPs was used to demonstrate one further SNP (aroE252G) that when interrogated discriminated ST93 from all other known STs [11]. For ST-93 discrimination the DNA template was quantified using UV spectrophotometry before use in PCR. This correlated with the 59,121,133 SNP profile termed “ST93 related” in this study [11]. All isolates were tested for the presence of the *pvl* gene [13].

For all bacteremic episodes, records were reviewed for the source of infection, the presence of systemic inflammatory response syndrome (SIRS) criteria on admission, intensive care (ICU) admission, comorbidities and survival at 30 days and 12 months. Community acquisition of infection was defined when the blood culture was drawn within 48 hours of admission, nosocomial if after 48 hours, and healthcare associated infection in those receiving dialysis, those admitted for 2 or more days in the preceding 90 days or nursing home residents.

Clinical and laboratory staffs were blinded to the results of molecular studies and clinical details respectively.

### Statistical analyses

Factors associated with *pvl* positive SAB strains were examined using univariable and multivariable logistic regression. Candidate predictors were identified for inclusion in the multivariable model using a combination of univariate performance (*p* < 0.10) and clinical relevance. A Hosmer and Lemeshow goodness-of-fit test was used to test the overall fit of the final logistic models. For all analyses, *p* < 0.05 was considered significant. All statistical analyses were performed using Stata version 11 (StataCorp, College Station, TX, USA). The study was approved by the Central Australian Human Research Ethics Committee. Individual informed consent was not required due to the observational nature of the study design.

## Results

Ninety-seven bacteremic episodes (20 pediatric, 77 adult) were identified in 94 individuals, 86 (91.4 %) of which were Indigenous.

MSSA accounted for 68.1 % of all bacteremia samples. On univariate analysis, MSSA was more likely community acquired (OR 2.50; 95 % CI 1.00, 6.22). All MRSA identified were of a nmMRSA phenotype and there was a trend for nmMRSA to be healthcare associated (OR 2.16; 95 % CI 0.86, 5.40,). The presence of SIRS criteria on admission, ICU admission, requirement for surgery and 30 day mortality did not differ by resistance phenotype.

*Pvl* positive strains accounted for approximately half of all episodes (54.3 %), regardless of resistance phenotype (Table 1). *Pvl* positive disease was associated with community acquisition (OR 5.48; 95 % CI 2.06, 14.62) and an SSTI focus in adults (OR 9.33; 95 % CI 2.37, 36.81). Among children there was a trend for *pvl* positive SAB to result from osteoarticular infections (OR 12.50; 95 % CI 0.80, 194.35). *Pvl* positive strains were also more likely to require surgical intervention (OR 6.93; 95 % CI 2.44, 19.66). Mortality did not differ between groups (Table 1).

**Table 1:** Patient characteristics of SAB episodes according to *pvl* positivity

	*pvl* Negative			*pvl* Positive			Total (94)	*p*-value
	n (%)			n (%)			n (%)	
	Adult (36)	Paed (7)	Total (43)	Adult (39)	Paed (12)	Total (51)		
Indigenous	30 (83.3 %)	7(100 %)	37 (86.1 %)	38 (97.4 %)	11 (91.7 %)	49 (96.1 %)	86 (91.5 %)	0.082
Age (range)			43.6 (0.42-95)			39.5 (3-76)		0.378
Male	22 (61.1 %)	3 (42.9 %)	25 (58.1 %)	25 (64.1 %)	8 (66.7 %)	33 (64.7 %)	58 (61.7 %)	0.514
RESIDENCE								
Remote	17/35^a^ (48.6 %)	5 (71.4 %)	22/42^a^ (52.4 %)	24 (61.5 %)	11 (91.7 %)	35 (68.6 %)	57 (61.3 %)	0.109
Town Camp	6/35^a^ (17.1 %)	1 (14.3 %)	7/42^a^ (16.7 %)	11 (28.2 %)	0 (0 %)	11 (21.6 %)	18 (19.4 %)	0.552
Alice Springs	10/35^a^ (28.6 %)	1 (14.3 %)	11/42^a^ (26.2 %)	3 (7.7 %)	1 (8.3 %)	4 (7.8 %)	15 (16.1 %)	0.017
Nursing Home/Prison	2/35^a^ (5.7 %)	0 (0 %)	2/42^a^ (4.8 %)	1 (2.6 %)	0 (0 %)	1 (2.0 %)	3 (3.23 %)	0.447
STAPHYLOCOCCAL TYPING								
MecA	9 (25.0 %)	2 (28.6 %)	11 (25.6 %)	13 (33.3 %)	4 (33.3 %)	17 (33.3 %)	28 (29.8 %)	0.413
ST93	1 (2.8 %)	0 (0 %)	2 (2.3 %)	20 (51.3 %)	4 (33.3 %)	24 (47.1 %)	25 (26.6 %)	<0.001
ST93 or related	1 (2.8 %)	0 (0 %)	2 (2.3 %)	30 (76.9 %)	9 (75.0 %)	39 (76.5 %)	40 (42.6 %)	<0.001
FOCUS OF INFECTION								
SSTI	4 (11.1 %)	1 (14.3 %)	5 (11.6 %)	21 (53.9 %)	1 (8.3 %)	22 (43.1 %)	27 (28.7 %)	0.001
Osteoarticular	5 (13.9 %)	2 (28.6 %)	7 (16.3 %)	5 (12.8 %)	10 (83.3 %)	15 (29.4 %)	22 (23.4 %)	0.134
Pneumonia	3 (8.3 %)	1 (14.3 %)	4 (9.3 %)	6 (15.4 %)	0 (0 %)	6 (11.8 %)	10 (10.6 %)	0.700
Endocarditis	3 (8.3 %)	0 (0 %)	3 (7.0 %)	0 (0 %)	0 (0 %)	0 (0 %)	3 (3.2 %)	0.055
Line Related	9 (25.0 %)	0 (0 %)	9 (20.9 %)	2 (5.1 %)	0 (0 %)	2 (3.9 %)	11 (11.7 %)	0.011
ACQUISITION								
Community Acquired	10 (27.8 %)	6 (85.7 %)	16 (37.2 %)	27 (69.2 %)	12(100 %)	39 (76.5 %)	55 (58.5 %)	<0.001
Healthcare Associated	25 (69.4 %)	1 (14.3 %)	26 (60.5 %)	12 (30.8 %)	0 (0 %)	12 (23.5 %)	38 (40.4 %)	<0.001
Nosocomial	9 (25.0 %)	1 (14.3 %)	10 (23.3 %)	4 (10.3 %)	0 (0 %)	4 (7.8 %)	14 (14.9 %)	0.037
SEVERITY								
SIRS	22 (61.1 %)	5 (71.4 %)	27 (62.8 %)	2 (74.4 %)	9 (75.0 %)	38 (74.5 %)	65 (69.2 %)	0.220
ICU admission	12 (33.3 %)	1 (14.3 %)	13 (30.2 %)	12 (30.8 %)	0 (0 %)	12 (23.5 %)	25 (26.6 %)	0.464
OUTCOME								
Surgery	7 (19.4 %)	2 (28.6 %)	9 (20.9 %)	21 (53.9 %)	12 (100 %)	33 (64.7 %)	42 (44.7 %)	<0.001
30 day Mortality	4 (11.1 %)	1 (14.3 %)	5 (11.6 %)	2 (5.1 %)	0 (0 %)	2 (3.9 %)	7 (7.5 %)	0. 156
12 month Mortality	10 (27.8 %)	1 (14.3 %)	11 (25.6 %)	5 (12.8 %)	0 (0 %)	5 (9.8 %)	16 (17.0 %)	0.055

Note: ST93 related includes ST93, ST59 and ST121

Abbreviations: *pvl* Panton Valentine Leucocidin gene, *SSTI* skin and soft tissue infection, *SIRS* systemic inflammatory response syndrome, *ICU* intensive care unit

^a^The residence for 1 adult patient was unable to be determined

Of the 54 nasal colonization isolates, 43 (79.6 %) were from Indigenous patients and 30 (55.6 %) MRSA. Only four isolates were *pvl* positive (7.4 %) and one was an ST93 related strain. None of the colonized patients developed bacteremia during duration of the study.

SAB isolates were significantly more likely to be *pvl* positive (OR 14.83; 95 % CI 4.27, 51.42) and belong to ST93 (OR 9.42; 95 % CI 1.99, 44.57) or ST93 related strains (OR 19.26; 95 % CI 3.81, 97.45) (Table 1). Nasal colonizing organisms were more likely to be MRSA than bacteremic organisms (2.95; 95 % CI 1.43, 6.01).

**Fig. 1:**
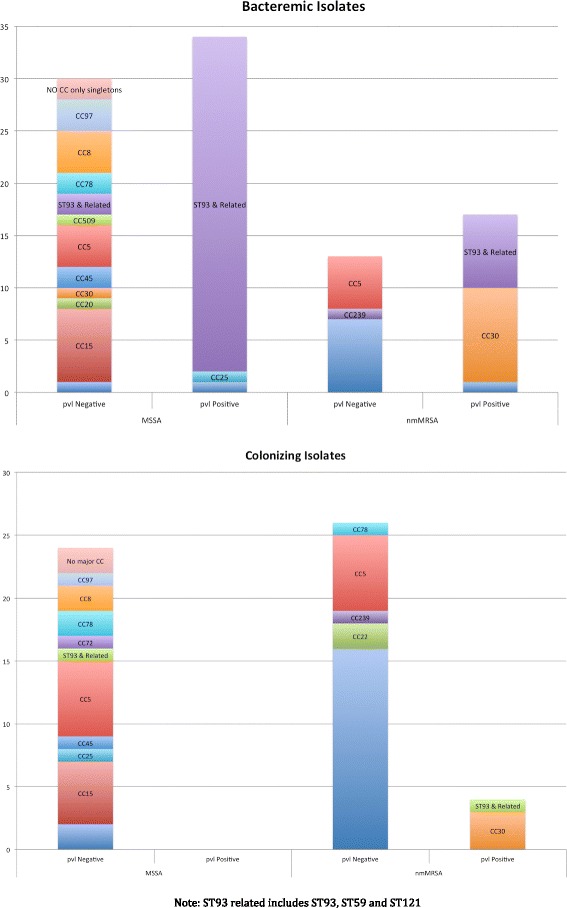
Clonal complex (CC) distribution of bacteremic vs colonizing isolates

The clonal complex (CC) distribution of bacteremic and colonizing isolates is shown in Fig. 1. In both cohorts, there was a broad range of strain types within the *pvl* negative MSSA group but a limited range in the *pvl* negative MRSA group. CC1, CC5 and CC239 were found in the *pvl* negative MRSA groups in both cohorts. The *pvl* positive ST93 related strains (CC 59/121/133) were the predominant strains found in MSSA bacteremia (53.1 %) and the second most common in MRSA bacteremia (23.3 %). Thirty-nine of 41 (95.1 %) bacteremic ST93 related isolates were *pvl* positive.

On multivariate regression, an identifiable focus of infection (OR 7.13; 95 % CI 1.28, 39.60), SSTI (OR 4.35; 95 % CI 1.16, 16.31) and community acquisition (OR 5.15; 95 % CI 1.85, 14.39) predicted *pvl* positive disease. Living in urban Alice Springs was associated with a lower chance of infection with a *pvl* positive strain compared with remote residence (OR 0.19; 95 % CI 0.04, 0.82).

## Discussion

Even within a geographically isolated continent, such as Australia, *S.aureus* exhibits remarkable variability in resistance patterns, virulence and epidemiology [2]. In Central Australia, we found a diverse range of MSSA sequence types, however, ST93 was clearly dominant and was strongly associated with *pvl* positivity, which was a risk factor for bacteremia following SSTI.

These findings are consistent with the aggressive clinical phenotype of ST93 and with recent Australian trends [14]. ST93 nmMRSA are frequently *pvl* positive, but recent research increasingly suggests that other virulence factors (alpha-hemolysin (Hla) exotoxin and alpha type phenol soluble modulins (α-PSMs)) may explain the increased virulence of ST93 staphylococcal infections [14]. In particular, over-expression of Hla - perhaps due to mutations in the regulatory gene *agr* and positive regulation by the *aryK* gene – may explain much of ST93 virulence [15].

ST93 CA-MRSA appears to be unique to Australia [2], although ST93 MSSA were present prior to their MRSA counterpart’s discovery. The coexistence of MSSA and MRSA ST93, with diversity even within the sequence type, has led to suggestions that the increasing rates of ST93 is not simply the result of clonal expansion of a single strain but rather perhaps by multiple different independent acquisitions of SCC*mec* in different ST93-MSSA strains [5, 16].

Similar to national trends, there is an increasing prevalence of CA-MRSA in Central Australia, 32 % in this study compared with 20 % earlier in the same decade [6].Reflecting trends elsewhere in Australia [17] and in the USA [18], CA-MRSA in Central Australia appears to be associated with healthcare contact.

Although resistance phenotype was not a virulence marker, *pvl* was associated with distinct clinical presentations. Among adults, *pvl* positive strains more often caused SSTI and tended to be associated with osteoarticular infections in children [3]. Reflecting the propensity for *pvl* positive strains to cause local tissue destruction [3], these strains were also more likely to require surgical intervention. Consistent with other studies, we found very few (10) pneumonic presentations, even in the setting of *pvl* [3]. Overall, thirty-day mortality was lower than expected, probably attributable to the surgical removal of the infective focus in the large number of SSTIs.

Sequence types of colonizing strains were similar for *pvl* negative MSSA and MRSA. However, very few *pvl* positive strains were found and only one ST93 isolate colonized the nasopharynx. The relative lack of *pvl* positive colonization has been reported previously [3, 19]. The different spectrum of colonizing to bacteremic strains questions whether individuals with nasal MRSA are at risk of invasive disease in our setting. Infection control strategies have relied on nasopharyngeal carriage as the principal reservoir for MRSA and colonization results in an increased infection risk [1]. However nmMRSA is known to colonize non-nasal sites including the axilla, groin and perineum and this is perhaps even more likely with *pvl* positive strains [3]. Apparent differences in the ecology of invasive and colonizing strains suggests that relying on nasal screening for hospital infection control measures will have limited success in Central Australia. Whether *pvl* positive organisms preferentially colonize non-nasal sites or are so highly virulent that they rapidly cause clinical infection requires further study.

Interpretation of colonization data is limited by potential selection bias because nasal MRSA screening typically occurs for patients admitted to intensive care. Consequently, the number of subjects for whom *S.aureus* colonization was determined was low. Moreover, as no colonized patients developed bacteremia, we cannot determine whether virulent *Staphylococci* also colonized the nasopharynx of clinically infected individuals.

## Conclusions

Our study further describes the expansion of nmMRSA, and the clinical impact of *pvl* positive *S.aureus*, particularly ST93, within an Indigenous population. The well documented rates of overcrowding, poor housing and poor access to hygiene infrastructure in Indigenous communities in Central Australia [20] are the perfect conditions to facilitate transmission of virulent *S.aureus* clones [21] and will likely contribute to increasing rates of nmMRSA and *pvl* associated disease in the future. The discrepancies in staphylococcal clones involved in bacteremia versus colonization warrant further investigation. Whether non-nasal carriage is more relevant or whether colonization with invasive clones occurs only transiently remains to be elucidated, but may impact on infection control practices in similar populations.

## Abbreviations

ASH, Alice Springs Hospital; CA-MRSA, community acquired methicillin-resistant *Staphylococcus aureus*; CC, clonal complex; HA-MRSA, healthcare associated methicillin-resistant *Staphylococcus aureus*; ICU, intensive care unit; MLST, multilocus sequence typing; MRSA, methicillin resistant *Stpahylococcus aureus*; MSSA, methicillin sensitive *Staphylococcus aureus*; nmMRSA, non-multiresistant methicillin-resistant *Staphylococcus aureus*; PCR, polymerase chain reaction; *pvl*, Panton-Valentine Leucocidin; SAB, *Staphylococcus aureus* bacteremia; SCC*mec*, staphylococcal chromosomal cassette; SIRS, systemic inflammatory response syndrome; SNP, single nucleotide polymorphism; SSTI, skin and soft tissue infection; ST, sequence type; STKP, single tube kinetic PCR
